# Audiobooks from terminally ill parent for their children – a qualitative evaluation

**DOI:** 10.1186/s12904-021-00872-6

**Published:** 2021-11-09

**Authors:** Henning Cuhls, Michaela Hesse, Gülay Ates, Lukas Radbruch

**Affiliations:** Department of Palliative Medicine, University Hospital Bonn, Sigmund-Freud-Str. 25, D-53127 Bonn, Germany

**Keywords:** Palliative care, Reminiscence, Biography, Bereaved children, Legacy

## Abstract

**Background:**

Improving the quality of life is one of the main objectives of palliative care. Biographical approaches are often used in combination with leaving a legacy in a range of different interventions such as Dignity Therapy or Life Review. This study presents an evaluation of audiobook biographies for palliative care patients with young children.

**Methods:**

Young parents diagnosed with a life-limiting disease could participate and create an audiobook for their young children. The audiobook itself was recorded over several days and edited by qualified radio journalists. After providing informed consent participants were interviewed twice over the course of the intervention regarding expectations, concerns, motivation, and experiences. Interviews and notes were transcribed verbatim and were analyzed using content analysis. The contents of the audiobooks are not part of the evaluation.

**Results:**

The data were collected from February 2017 till September 2020. Fifty-four patients with ninety-six children at a mean age of 7 years were included and created an audiobook. The main theme of all interviews were the children. Within this field identified main topics were legacy, motivation, usage, benefit, aims, difficulties and worries in descending order. All patients would recommend the intervention.

**Conclusion:**

Creating an audiobook as a legacy to their children seemed to help the diseased parents to cope with their limited life span.

## Background

Existential suffering [1], related to anxiety, mental anguish and psychosocial suffering, is frequent in patients with a life-threatening disease and requires spiritual and psychosocial support for these patients. A range of interventions has been described in palliative care to cover psychosocial needs and to relieve the distress of patients. There is a growing interest in biographical approaches with the creation of legacy documents, including interventions such as Dignity therapy, Life Review, or Reminiscing [2]. Biography interventions can offer a coping strategy with the creation of a life narrative and a legacy for the loved ones [3]. Keall et al. found in their review that legacy activities have positive effects on patients’ level of interaction [4]. Reviews of interventions with a biographical approach confirmed a reduction of depression and improvement of quality of life overall different methods and study designs [5–8]. Leaving young children behind can be assumed to be an additional burden for patients with life-threatening diseases, aggravating distress in the dying process. In Germany every year 37,000 parents with young children find themselves confronted with a cancer diagnosis [9]. Audiobooks as a legacy for the children might alleviate this distress. This study was initiated to monitor the outcome and effectiveness of an audiobook intervention. Funding from the RheinEnergieStiftung (No. F-16-2-11) covered the costs of the audiobook creation so that patients got it free of charge.

## Methods

This section is following the instructions of the TIDieR checklist [10]. The overall aim of this study is an evaluation of an audiobook intervention for terminally ill parent with young children by qualified radio journalists. Participants were interviewed before the first and after the last recording to examine the appropriateness and fulfilment of expectations, concerns, motivation, and experiences. So, in case of distress, a mismatch or inappropriate crossing of boundaries, the intervention can be reflectively revised and adjusted within the team to meet the needs of participants. The study was designed as a mixed-methods approach with questionnaires and semi-structured interviews twice in the run of the creation of the audiobook. The quantitative analysis will be described separately.

All methods were carried out following relevant guidelines and regulations such as COREQ [11], GCP and the Declaration of Helsinki. The research ethics committee of the University Hospital Bonn reviewed and approved the study (no.389/16).

### Setting/participants

In the first years (2017–2019) recruitment for the study was restricted to patients from Northrhine-Westphalia due to funding specifications of the RheinEnergieStiftung. In 2020 recruitment restrictions were lifted and patients could be included from all over Germany, Austria and Benelux. Participants were recruited by a social worker, a nurse or a psycho-oncologist with research training. Due to high media attention to the project, most of the patients asked for participation themselves. Patients had to sign a self-disclosure form about their case history. Inclusion criteria were checked and patients were included following written informed consent. None of the interviewed patients was treated by members of the recruitment or interviewer team.

Inclusion criteria for this study were (1) patients with a short remaining life expectancy, (2) fluent in the German language, (3) 18 years of age or older and (4) having at least one child younger than 18 years. The exclusion criterion was psychiatric impairment such as dementia, psychosis, severe depression or diagnosed personality disorder.

### Provider

The process of making an audiobook is complex and contains narratives, songs performed by the patients such as lullabies, favourite or important pieces of music, readings and other material patients wanted to include. The recording took place according to the patients’ wishes in the hospital, at patients’ homes, in a flat in Cologne or a small Eifel town apartment. In 2020 modifications were necessary due to Covid-19 and led to a completely digital process of recording. Patients were equipped with a special microphone by mail and had to download a program on their computer. The duration of recording could be from a few hours up to 3 days.

In the years 2017–2019 interviews were led by Judith Grümmer. Since the end of 2019, the interviews were led by another eight radio journalists with special training. Application for this audiobook training could be made by journalists experienced in interviewing and editing. Sixteen journalists were selected and trained in a 40 h course with subsequent supervised practical training. The course included knowledge about life-threatening illnesses, goals and basics of palliative care, interviewing, role-play, self-experience and supervision. Eight journalists started in the project. The training course was conducted by the palliative care academy at the Helios Hospital Bonn and funded by RheinEnergieStiftung. All journalists were trained in self-care management and had a 24/7 main contact person to deal with emotional and psychological stresses related to the reception situation [12].

### Data collection

Participating patients were assessed with semi-structured interviews before and after the audiobook recording. Semi-structured interviews included questions such as “What is your expectation?”, “What are your main concerns?”, “What is your motivation?”, and feedback questions such as “How did you experience the intervention?” and “Would you recommend the audiobook?”. Further questionnaires assessed the degree of quality of life and the importance of specific areas in life. The qualitative and quantitative data capture knowledge at different levels, that cannot easily be paralleled in biography work. Within this paper, we focus on the qualitative data material, which in itself has a very high intrinsic value. The spoken word with its richness and detail gives particular insights into motivation, meanings and importance of the biographical work of each individual.

The interviews were shorthanded or audiotaped, and additional notes were taken. Interviews were performed face-to-face or by telephone depending on health status and restrictions from Covid-19. Patients were interviewed by an experienced and trained biographical researcher and physician specialized in the field of palliative care (HC physician and MH psycho-oncologist).

### Analysis

The interviews and notes were transcribed verbatim and transcripts were analysed using content analysis with the software MAXQDA-18.

First codes were generated deductively from the questions. Also, the entire data set was checked systematically for generating codes inductively. Relevant data were collected for each code. Two of the authors (MH and HC) assured that no new codes were derived from the data. MH and HC reviewed codes independently checking the entire data set for generating a codebook. Differences in coding were discussed until consensus was reached. Intercoder reliability [13] was demonstrated twice during the coding process. All consolidated criteria for reporting qualitative research (COREQ) were considered [11].

## Results

The data were collected from February 2017 till September 2020. Fifty-eight patients were included. Four patients dropped out due to a declining performance status or death after their inclusion. Another ten patients agreed to participate but became too weak or died before informed consent. The second interview was not possible for 28 patients due to health status decline or death. The analysis encompassed qualitative data independently of a second-time point for an evaluation of the intervention or the length of completed audiobooks. The sociodemographic characteristics are shown in Table 1.

**Table 1:** Sociodemographic characteristics

Gender	
Men	*n* = 13, mean age 43,9 (SD 33–57)
Women	*n* = 41, mean age 41,7 (SD 35–57)
Martial states	
Married/living with a partner	*n* = 45
Divorced	*n* = 7
Single	*n* = 2
Cancer	*n* = 50 [gynecologic (*n* = 23), gastrointestinal (*n* = 13), brain (*n* = 5), lung (*n* = 2), other (*n* = 7)]
Non-cancer	*n* = 4 (1xCOPD Stage IV, 2x Amyotrophic lateral sclerosis, 1x Huntington’s disease)
Children	*n* = 96, mean age 7,25 (SD 0.4–18) discordant value: 1 handicapped child 27 years old

### Findings

The predominant topic in all interviews were the children. Within this topic legacy, motivation, usage, benefit, aims, difficulties and worries were identified as major categories in descending order. The distinction between categories was sometimes difficult because patients’ statements were complex. The quotes encompassed more than one aspect such as “It is very important that you can listen to this audiobook in peace and listen to the whole story, the life story, so to speak, of dad and me over the years, to perhaps draw strength from it”. (ZBE15) We discussed this in depth and decided to code this passage double with categories motivation and aims, and in this case even triple with usage.

Patients described their experience in the recording sessions as exhausting but were at the same time satisfied and happy about all the memories that came up. The categorization is explained in Table 2: Codebook.

“Above all, I made this book for my children [ … . ] but it was also important for me to engage with my life intensively. A lot of sweet memories appeared and I could clear the air with some bitter experiences. The recording days were an emotional roller coaster ride. I cried time after time, but I laughed a lot.” (ZE9f)

“I started to talk about my grandparents, my parents, my childhood, all the things I experienced and I mentioned that I had a good life so far. I have a lot of positive memories and beautiful experiences. It is exhausting to tell all that and you have all these memories but it made me so thankful.” (CW214)

**Table 2:** Codebook

Category	Definition	Codes
Legacy	Everything described to be passed to the children and kept for their future	generation, preserve, reminiscence, leave behind, advice, life story, connection, treasure, guidance
Motivation	intentions and reasons for taking part	first-hand information, self-reflection, personal gain, companion, help
Usage	how to deal with the audiobook	single chapter vs. completely, reference book, listen in company, some chapters later when the children are older
Benefit	the positive impact of the intervention	gift, gratitude, affecting time, well-being
Aims	what patients want to achieve	availability on demand, pleasure, orientation, source of strength, sweet memories
Difficulties	Obstacles in the creation of the audiobook	adaptation to interview, coping, telling of bitter memories, the complexity of memories, authenticity, finding right wording for children, consideration, overcoming
Worries	Everything mentioned what parents were afraid of	point in time to listen to single chapters, bitter memories, grief, challenge, truth and awareness

All patients would recommend the audiobook to others and would do it again. This did not change when the recording had to be digital due to Covid-19.

“I was sitting in front of my laptop which was equipped with a special program. I could see the biographer. I took notes beforehand. Once I got started a lot of things came into my mind that I wanted to tell. (CW3 213)

Illustrative quotes were chosen to exemplify our findings.

Patients described obstacles in the creation of the audiobook (category difficulties), such as staying authentic:

*„I don’t want to sweep anything under the carpet. I would like my child to see me as I am, how I feel, where I came from and where I’ve been.“* (S1E5)

A parent described what they were afraid of (category worries), such as grief:

*„I can imagine that hearing my voice after I’m dead maybe will be a shock for my children. But hopefully, in time, it’ll reward them and be a source of happiness and reassurance.“* (B1FM5)

The intervention had a positive impact (category benefit), such as well-being:

*„After recording it, I’ve had intense talks with family members that could only have taken place with the audiobook as support. I note that my fear of the end, of death, hasn’t disappeared, but it has become a little smaller. The audiobook lets me find peace, so I can concentrate more on the here and now.* “(BBD19).

Patients described what they want to achieve (category aims), such as a source of strength:

*„It’s very important that my children can take their time to listen to this audiobook, and to hear bit by bit the whole tale, actually the life story of daddy and me, and listen to it again and again over the years. Perhaps they can draw some strength from it. “*(Z1B5)

The question of how to deal with the audiobook (category usage) was very important, such as the idea that some chapters should be heard when the children are older:

*„And possibly, depending on how old they are when they listen to it, they will notice different things. They should only listen to the chapter about my illness when they are a little older. “*(G2M8)

Participants described their intentions and reasons for taking part (category motivation). It was important for them to give first-hand information:

*„It’s important for me to do this so that my child can maybe learn something about his father’s life. So that a combination of my voice and the stories will perhaps give him a feeling of how his father really was. “*(M3L2)

Parents wanted to leave something behind (category legacy), such as reminiscence and guidance:

*„This audiobook gives them the chance to hear my voice whenever they want. Also for a long time after I’m no longer there. I’m very happy to leave this possibility with them. Perhaps the audiobook won’t be just a memory, but rather a beam of light that can lead them through dark times. “*(V3M1)

## Discussion

Many patients took a long time to initiate the recording of the audiobook though they had had all information for several months before, but oncological therapy, the family or other priorities might have been more important. Some already had a bad performance status due to disease progression when they got started. In consequence, the second assessment after the recording was not possible for all participants. The dropout rate was in the expected range in a palliative care setting [9, 14–16]. More women than men completed an audiobook in our study, as the majority of patients dropping out before or after inclusion were men (10 out of 14). This suggests that men waited longer with the realization of the audiobook than women.

In a review of biographical interventions in palliative care, we found several different approaches with written legacy documents [2]. However, there has been no research on oral legacy such as audiobooks. The mean age of the patients’ children was 7 years so that it may be assumed that the majority was not able to read a written document. Preserving the voice of the mother or father offers additional qualities because it transports a lot of personalities. Temper, humour, laughter, special and unique phrases, or idioms were recorded and children might be able to get a feeling of the character of their parents. Lindquist et al. discussed the impact of the editor on the patients’stories [17]. The audiobook requires less editing than the production of a written document from a transcript of an interview. The recorded material was cut but the stories were not changed. The chance to equalize the voice in case of wheezing for example was utilized. The recipients of the stories in this project were the children and the participants put less focus on the biographer as discussed by Lindquist. The intervention was aimed at patients with small children. Most patients had already thought about the content of their audiobook and had ideas about what they wanted to tell their children. It comes as no surprise then that the evaluation confirmed the children as the predominant theme of the project. This is in contrast with a previous study on a biography intervention with older patients, where the main interest was in leaving a legacy [18]. This difference corresponds with a study from Ando et al. exploring the primary concerns of advanced cancer patients in a life review intervention [19]. They found differences in concerns depending on age, with patients in the 40-year-old group mainly interested in their children.

Biography interventions can offer a coping strategy with the creation of a life narrative and a legacy for the loved ones. Keall et al. found in their review that legacy activities have positive effects on carers’ level of stress and patients’ level of interaction [4].

Participants wanted to leave information, consolation, and advice for their children but did not expect a benefit from the intervention for themselves. We know from research that interventions comprising biographical elements are effective in increasing spiritual well-being and decreasing depression. Reminiscing had social, instrumental, and integrative functions and seemed to foster coping in a study by Westerhof et al. [20]. They concluded that reminiscing had an impact on psychological resources such as social support, mastery, coping, meaning in life and self-esteem. The findings in our interviews confirm this effect. Parents were very much concerned about the usage of the audiobook. They were worried about the extent, content, and who could accompany the children in the usage of the audiobook. Some participants wanted parts of the audiobook to be separated and only be used by the children they were older. The idea to record today and address their children in 10 or 20 years was challenging. Some parents struggled with the conflict of being authentic and the degree of accuracy of bitter memories. This problem was already discussed by Lindquist et al. who assumed that biographical interventions might be co-constructing a picture of a person with means of over idealization [17].. We found a lot of parents who were very reflective of their impact and influence in this recording process. Especially those who were divorced or living apart restricted themselves and did not talk in a bad manner about the ex-spouse. On the contrary, they wanted to bridge the gap to the surviving parent.

Further research should focus on the usage and the impact of these audiobooks on bereaved children. All human beings ask for their origin and want to know about their parents. To lose a parent has a high impact on the whole life of young children and it may well be possible that the audiobook can prevent children from becoming depressed or suffering from a traumatic disorder.

### Study limitations

The period between the evaluations and recording phase was very broad related to organizational issues. We had little insight into the interview and the recording process which was performed in a separate setting with the professional journalists. In 2020 the interaction and exchange with patients were limited due to Covid-19. Nevertheless, we found a high motivation to participate in the evaluation. Some participants even wrote an email reporting their experiences with the recording. A further limitation is due to translation and some data were transcribed interviews versus shorthand notes.

We had no patient and public involvement, this should be incorporated in future studies.

## Conclusion

Although there are some limitations, our study emphasized the benefit of the biographical intervention. Creating an audiobook seemed to help the parent to cope with their limited life span. The possibility to leave a legacy to their young children was a great relief despite the huge effort. Audiobooks are a way to preserve a connection with very young children. The audible affirmation of eternal love might be expected to help in growing up without the birth mother or father.

## Data Availability

The recorded datasets, transcripts and audiobooks are sensitive information and underly the personality rights of the patients. We have written consent to use the material for this study, but we are not allowed to share this not blinded material publicly. Further Information can be obtained from the corresponding author.
